# The Histidine Biosynthetic Genes in the Superphylum Bacteroidota-Rhodothermota-Balneolota-Chlorobiota: Insights into the Evolution of Gene Structure and Organization

**DOI:** 10.3390/microorganisms9071439

**Published:** 2021-07-03

**Authors:** Sara Del Duca, Christopher Riccardi, Alberto Vassallo, Giulia Fontana, Lara Mitia Castronovo, Sofia Chioccioli, Renato Fani

**Affiliations:** Department of Biology, University of Florence, Via Madonna del Piano 6, Sesto Fiorentino, 50019 Florence, Italy; sara.delduca@unifi.it (S.D.D.); christopher.riccardi@unifi.it (C.R.); alberto.vassallo@unifi.it (A.V.); giulia.fontana1@stud.unifi.it (G.F.); l.castronovo@student.unisi.it (L.M.C.); sofia.chioccioli@unifi.it (S.C.)

**Keywords:** gene duplication, gene fusion, operon origin, operon evolution, regulons

## Abstract

One of the most studied metabolic routes is the biosynthesis of histidine, especially in enterobacteria where a single compact operon composed of eight adjacent genes encodes the complete set of biosynthetic enzymes. It is still not clear how *his* genes were organized in the genome of the last universal common ancestor community. The aim of this work was to analyze the structure, organization, phylogenetic distribution, and degree of horizontal gene transfer (HGT) of *his* genes in the Bacteroidota-Rhodothermota-Balneolota-Chlorobiota superphylum, a group of phylogenetically close bacteria with different surviving strategies. The analysis of the large variety of *his* gene structures and organizations revealed different scenarios with genes organized in more or less compact—heterogeneous or homogeneous—operons, in suboperons, or in regulons. The organization of *his* genes in the extant members of the superphylum suggests that in the common ancestor of this group, genes were scattered throughout the chromosome and that different forces have driven the assembly of *his* genes in compact operons. Gene fusion events and/or paralog formation, HGT of single genes or entire operons between strains of the same or different taxonomic groups, and other molecular rearrangements shaped the *his* gene structure in this superphylum.

## 1. Introduction

The origin and evolution of metabolic pathways represent one of the most crucial events that occurred during molecular and cellular evolution [1], since they rendered the primordial cells less dependent on the exogenous supply of abiotically formed molecules. This issue is often linked to the origin and evolution of operons [2] and can be studied through either directed evolution experiments and/or the comparative analysis of genes involved in the same metabolic pathway. One of the most studied metabolic routes is the biosynthesis of histidine, which has been extensively studied in *Salmonella enterica* and *Escherichia coli* [3]. In these enterobacteria, a single compact operon composed of eight adjacent genes (*hisGDC[NB]HAF[IE]*) encodes the complete set of histidine biosynthetic enzymes. Three of the eight genes (*hisD*, *hisNB*, and *hisIE*) encode bifunctional enzymes, while two (*hisH* and *hisF*) encode a heterodimeric enzyme catalyzing a single biosynthetic step, for a total of 10 enzymatic reactions [4].

This pathway represents an important metabolic crossroad since it is intimately connected to (at least) two important metabolic routes: nitrogen metabolism and de novo synthesis of purines. This interconnection is due to the activity of imidazole-glycerol-phosphate synthase (IGPS), a heterodimeric enzyme composed of the glutaminase subunit HisH and the cyclase subunit HisF, and catalyzing the fifth step of the L-histidine biosynthetic pathway [3]. The reaction catalyzed by IGPS produces both imidazole-glycerol-phosphate (IGP) and 5-aminoimidazole-4-carboxamide ribonucleotide (AICAR or ZMP). AICAR is recycled into the de novo purine biosynthetic pathway, while IGP is dehydrated by an activity of a bifunctional enzyme, encoded by *hisNB*. In addition to this, the two initial substrates of histidine biosynthesis, PRPP and ATP, play key roles in intermediary and energy metabolism and link this pathway to the biosynthesis of purines, pyrimidines, pyridine nucleotides, folates, thiamine, and tryptophan [4,5].

The importance of histidine biosynthesis in bacterial metabolism is also underlined by the ancestry of this metabolic pathway. Indeed, there are several independent pieces of evidence for the antiquity of the histidine biosynthesis pathway. The chemical synthesis of histidine and of histidyl-histidine under primitive conditions has been reported, as well as the role of the latter in the enhancement of some possible prebiotic oligomerization reactions involving amino acids and nucleotides [6,7]. It has also been suggested that histidine may be the molecular vestige of a catalytic ribonucleotide that existed in the RNA world [8,9]. If histidine was required by the first catalytic molecules, then the eventual exhaustion of the prebiotic supply of nutrients might have imposed a (strong) selective pressure favoring those primordial living beings that became able to synthesize the amino acid by themselves. Thus, it is very likely that the entire pathway was assembled long before the appearance of the last universal common ancestor (LUCA) of the three extant cell domains, as suggested by the analysis of several archaeal, bacterial, and eukaryotic completely sequenced genomes, and that different molecular mechanisms have been responsible for the evolution of *his* genes [1,7]. Indeed, in some species, more than one enzymatic function is encoded by the same bi- or multifunctional cistron, such as *hisD*, *hisNB*, *hisHF*, and *hisIE* in some prokaryotes and HIS4 and HIS7 in eukaryotes [3]. These multifunctional genes are the outcome of more or less ancient fusion events. Moreover, gene duplication and gene elongation also played a key role in shaping histidine biosynthesis, such as in the case of *hisA* and *hisF* paralogs, which represent a paradigmatic example of how complex genes can be constructed by simpler ones [3,10].

### 1.1. Organization of the Histidine Genes

It is still not clear how *his* genes were organized in the genome of the LUCA community. However, Fondi et al. [9] suggested the absence of a complete histidine operon in LUCA and that (at least some) *his* genes were scattered throughout its genome. After the assembly of the entire pathway, the organization of *his* genes underwent major rearrangements in the three domains, generating a wide variety of structural and/or clustering strategies of *his* genes, with organisms showing scattered genes, partially compacted in sub-operons or organized in more or less compact operons [11,12]. Hence, several *his* operons appear to be the result of recent events of evolution and were very likely constructed by a “piecewise” mechanism [12,13]. The model was originally suggested to explain the origin and evolution of the proteobacterial operon and predicts that *his* genes were scattered on the genome of the proteobacterial ancestor. All these genes (except for *hisD*) coded for monofunctional enzymes and underwent a progressive clustering through the formation of three suboperons (*hisGDC*, *hisBHAF*, and *hisIE*). Then, these suboperons joined to form a single unit, and clustering culminated in some **γ**-proteobacteria (e.g., *E. coli* and *S. enterica*) where the operons are very compact and include fused and/or overlapping genes. The entire operon (or parts thereof) was then horizontally transferred to other microorganisms belonging to the same or to different phylogenetic branches [3]. Such a mechanism might have also acted in the assembly of some Archaeal *his* operons [9].

### 1.2. Regulation of the Histidine Biosynthesis

The considerable metabolic cost (41 ATP molecules are consumed for each histidine molecule made) accounts for the evolution of complex strategies in different organisms to finely modulate the rate of synthesis of this amino acid in response to environmental changes. In *S. enterica* and in *E. coli*, the biosynthetic pathway is under the control of distinct regulatory mechanisms that operate at different levels [14], including (i) histidine-mediated feedback inhibition of the activity of the first enzyme of the pathway (i.e., HisG) almost instantaneously, which adjusts the flow of intermediates along the pathway in response to the availability of exogenous histidine, and (ii) transcriptional attenuation at a regulatory element, located upstream of *hisG*, which allows coordinate regulation of the levels of the histidine biosynthetic enzymes in response to the variations of histidyl-tRNA. Moreover, (iii) transcription of the *his* operon is also modulated at the level of intra-cistronic Rho-dependent terminators by a nonspecific mechanism operating during the elongation step. Terminators account for the polarity exhibited by several nonsense and frameshift mutations. Very few studies of the regulation of *his* operon expression in this area have been performed with other prokaryotes [4]. However, it has been shown that in several bacteria, the regulation of histidine biosynthesis is mainly due to the feedback regulation of HisG. The inactivation of HisG activity in the presence of histidine involves HisZ, which is able to bind and inactivate HisG enzyme [15]. Thus, it appears that different *his* gene structural, organizational, and regulatory strategies are present in diverse bacteria. However, it is not still clear whether the different gene structure and/or organization are more closely linked to phylogeny or to the ecological niche inhabited by different bacteria.

### 1.3. The Bacteroidota-Rhodothermota-Balneolota-Chlorobiota Superphylum

An example of phylogenetically close bacteria exhibiting very different surviving strategies and inhabiting a wide variety of environments is the Bacteroidota-Rhodothermota-Balneolota-Chlorobiota superphylum. Bacteroidetes, previously known as the Cytophaga-Flavobacteria-Bacteroides (CFB) group [16], inhabit many different ecological niches; they have colonized many habitats, including soil, ocean, freshwater, the oral cavity of humans, and the gastrointestinal tract of animals, where they display various biological functions [17,18]. Indeed, they show a high metabolic diversity within the phylum, including aerobes and anaerobes, even though Bacteroidetes are generally chemoorganoheterotrophs [19]. In contrast to the wide distribution of Bacteroidetes species in diverse habitats, bacteria from the phylum Chlorobi (also commonly known as green sulfur bacteria) occupy a narrow environmental niche mainly consisting of anoxic aquatic environments, where sunlight can penetrate [20], and they are all obligately anaerobic photoautotrophs [21]. Although Bacteroidetes and Chlorobi are presently recognized as two distinct phyla, these two groups appear to be closely related, on the basis of 16S rRNA and other gene sequences and the presence of conserved indels that are uniquely found in species from these two groups [17]. Recently, Bacteroidetes and Chlorobi were suggested to belong to the “Bacteroidota-Rhodothermota-Balneolota-Chlorobiota superphylum” [22,23]. Rhodothermota and Balneolota are both recently proposed phyla, former members of the phylum Bacteroidetes [24,25].

The phylogeny of Bacteroidetes has been extensively studied for years, and the rapid increase of the taxonomic complexity of the phylum encouraged the revision of its phylogeny [22,26,27]. However, this classification has proven to be difficult because of insufficiently resolved 16S rRNA gene trees or incomplete taxon sampling. Recently, a work was published on the genome-based taxonomic classification of more than 1000 members of the superphylum and outgroup type strains [19].

Thus, the aim of this work was to analyze the structure, organization, phylogenetic distribution, and degree of horizontal gene transfer of *his* genes in this group of phylogenetically close bacteria (but with different lifestyles) in order to shed additional light on these issues and on the validity of the piecewise construction of histidine biosynthetic operons.

## 2. Materials and Methods

### 2.1. Sequence Data Source and Sequence Alignment

Ninety-one representatives of the four phyla (9 Chlorobiota, 4 Balneolota, 6 Rhodothermota, and 72 Bacteroidota) listed in Table S1 (Supplementary File S1) and 17 outgroups listed in Table S2 (Supplementary File S1) were considered eligible for the purpose of this work, resulting in a total of 108 organisms. Histidine biosynthetic enzyme sequences of the selected strains were retrieved manually from UniProtKB [28]. A data frame was then built by pasting the accession numbers of the GenBank complete genomes assemblies and the locus tags of the histidine biosynthetic proteins (Tables S1 and S2, Supplementary File S1). All genomes were downloaded from the NCBI and a BLAST database constructed through Galaxy Sever (Galaxy Version 2.10.1 + galaxy0) [29,30]. Local BLASTs were launched on a Galaxy Server environment.

His proteins amino acid sequences were aligned using BioEdit [31] through the ClustalW tool [32] to evaluate the identity/similarity between amino acid sequences, which have been calculated through BLOSUM62 similarity matrix.

### 2.2. Phylogenetic Trees Construction

All multiple sequence alignments (MSA) used for phylogenetic tree construction were built using MAFFT [33]. The resulting MSAs were further processed through trimAl [34], using its option “automated1”, which provides a heuristic selection of the automatic method based on similarity statistics and is optimized for maximum likelihood (ML) phylogenetic tree reconstruction. After filtering, each MSA was forwarded to MEGA7 [35] for phylogenetic analysis with 100 bootstrap replicates and a thorough ML search.

In order to gain a deeper insight into the evolutionary relationship among the species under study, a set of orthologous genes were retrieved using a bidirectional best hit (BBH) approach. As the BBH entails identifying the pairs of genes in two different genomes that are more similar to each other than either is to any other gene in the other genome, a pairwise bidirectional search was performed between every gene present in *Bacteroides fragilis* NCTC 9343 (one of the 91 ingroups, belonging to Bacteroidota) and the proteomes of each of the 108 organisms under investigation, using in-house Python scripts (available at https://github.com/chrisondakeys/pairwise_bbh, accessed on 22 June 2021) and BLAST+ executables [29]. Once the suitable orthologs were found, in order to have a general overview of the functional category they best associate with, these were uploaded to eggNOG-mapper webserver [36] in the form of a multi-FASTA for inferring the clusters of orthologous group (COG) categories represented within this pool.

Genomic organization of *his* genes has been retrieved from NCBI using the graphics option. Information on the organisms’ growing temperature range has been obtained from the Bacterial Diversity Metadatabase BacDive [37].

## 3. Results and Discussion

### 3.1. Retrieval of His Biosynthetic Protein Sequences from Bacteroidota, Chlorobiota, Balneolota, and Rhodothermota and from Outgroups

On the basis of the work of García-López et al. [19], we selected a panel of representatives of the phyla Bacteroidetes, Chlorobi, Balneolota, and Rhodothermota. For Chlorobi, Balneolota, and Rhodothermota, all the organisms considered in their work (i.e., 10, 7, and 9, respectively) were considered. For Bacteroidetes, a representative for each main group of the phylogenetic tree obtained by García-López et al. was chosen.

Histidine biosynthesis protein sequences were downloaded from UniProtKB. Those organisms whose sequences were not available on UniProtKB were discarded. For Bacteroidetes, when histidine proteins of a chosen representative were not available on UniProtKB, a new representative of the same phylogenetic group was selected. In this way, 91 representatives of the 4 phyla (9 Chlorobiota, 4 Balneolota, 6 Rhodothermota, and 72 Bacteroidota) listed in Table S1 (Supplementary File S1) were considered.

Histidine biosynthesis protein sequences were also downloaded for 17 outgroups belonging to different bacterial taxonomic groups listed in Table S2 (Supplementary File S1).

### 3.2. Structure of Histidine Biosynthetic Genes in Bacteroidetes, Chlorobi, Balneolota, and Rhodothermota

It has been previously shown that several histidine biosynthetic genes underwent different molecular rearrangements, such as elongation, duplication, and/or fusion events, in different phylogenetic lineages. In order to check if such events might also have occurred in this bacterial group and for the presence of unusual structural gene rearrangements, the structure of His protein sequences from each of the 91 strains was analyzed.

#### 3.2.1. Structure of *hisA*/*hisF*

The *hisA* and *hisF* gene products code, respectively, for a N’-[(5′-phosphoribulosyl)-formimino]-5-aminoimidazole-4-carboxamideribonucleotide (5′-ProFAR) isomerase catalyzing the fourth step of the histidine biosynthetic pathway and for a cyclase catalyzing the fifth step, forming the heterodimer IGPS with the glutamine amidotransferase subunit HisH [5].

It is known [11,38] that the two genes *hisA* and *hisF* have a common ancestry and are the result of a cascade of at least two duplication events, involving an ancestral gene half the size of the present-day ones. This gene underwent a first elongation event giving rise to the ancestor of *hisA* and *hisF*. This in turn duplicated, generating *hisA* and *hisF*. Del Duca et al. [10] also suggested that the two genes could be the results of a cascade of gene elongation/domain shuffling events starting from an ancestor gene coding for a module one eighth the size of the present-day ones. Hence, the HisA and HisF sequences were retrieved from the genome of the 91 representatives, multialigned, and analyzed.

The analysis of the selected HisA and HisF sequences (Multialignment, Supplementary Files S2 and S3) revealed a slight difference in the length of HisA proteins between Bacteroidetes and Chlorobi-Rhodothermota: indeed, the Chlorobi-Rhodothermota HisA sequences are longer than those of the Bacteroidetes; this is due to the addition of a short peptide sequence at the *C*-terminal region of HisA. The same additional *C*-terminal region is shared by Chlorobi (except for *Ignavibacterium album* JCM 16511 and *Melioribacter roseus* P3M-2, which have a shorter HisA sequence similarly to Bacteroidetes) and Rhodothermota, suggesting that the *I. album* JCM 16511 and *M. roseus* P3M-2 *hisA* genes might have been acquired via horizontal gene transfer (HGT), an idea that is in agreement with the phylogenetic trees constructed using the amino acid sequence of the His proteins (see Section 3.3).

The analysis of the length of HisF revealed a higher conservation than that found in HisA. It is possible that this conservation might be due to functional and/or structural constraints, since HisF interacts with HisH to give a functional heterodimeric IGPS (Ref. [3] and reference therein). This also agrees with the finding that the degree of sequence identity/similarity is higher between HisF orthologs than those of HisA (not shown).

The comparative analysis of the HisA and HisF amino acid sequences of all the analyzed Bacteroidetes, Balneolota, Rhodothermota, and Chlorobi revealed a high degree of sequence similarity and that the two proteins shared the two-paralogous module structure (not shown). Moreover, we also checked the similarity existing between modules of HisA/HisF proteins; indeed, it is known that the two proteins share a TIM-barrel tertiary structure, consisting of eight (β/α) modules (each of which has about 30 residues) [39]. The analysis of the HisA and HisF sequences revealed that some of them exhibit a length of exactly 240 amino acids. Hence, we split these sequences into eight 30-amino-acid-long modules and checked the degree of sequence identity/similarity between them. As an example, data obtained in the case of HisA modules of *Dyadobacter fermentas* DSM 18053 are reported Table 1.

The analysis of data reported in Table 1 revealed that the eight modules shared a degree of sequence similarity sufficiently high to suggest their possible common origin from an ancestor module, strongly supporting the cascade of three gene elongation events suggested by Del Duca et al. [10].

#### 3.2.2. Structure of *hisB* and Identification of a *hisN* Homologue

The sixth and the eighth steps of histidine biosynthesis are catalyzed by histidinol-phosphate phosphatase (HOL-Pase) and imidazole-glycerol-phosphate dehydratase (IGPD), respectively [40]. Different phosphatases can perform the dephosphorylation of HOL-P in different organisms, whereas the dehydration of IGP is carried out by the same enzyme in all histidine-synthesizing organisms [41,42]. In *E. coli*, the two activities are encoded by the *hisNB* bifunctional gene [41], which is formed by two regions, i.e., a proximal one (*hisN*) coding for the phosphatase moiety of the DDDD-type and a distal one (*hisB*) encoding the dehydratase activity. The likely evolutionary pathway of *hisNB* [3,41] predicts that this bifunctional gene is the outcome of a fusion event of two separated cistrons (*hisN* and *hisB*) coding for a HOL-Pase and an IGPD, respectively, and occurred in the **γ**-branch of the proteobacteria. The *hisNB* gene might have then been horizontally transferred from **γ**-proteobacteria to other microorganisms within the entire *his* operon (*GDC[NB]HAF[IE]*) or parts thereof [3,12].

In order to check the structure of *hisN*-*hisB* genes in the members of the superphylum, firstly, all the HisB amino acid sequences retrieved were aligned. The entire multialignment is reported in the Supplementary File S4. Data obtained revealed that all the Chlorobi (except for *M. roseus* P3M-2) and Rhodothermota HisB protein sequences analyzed correspond to the 3′ region of the *E. coli* bifunctional gene, coding for an IGPD domain. On the contrary, in the genome of all the considered Bacteroidetes, we found a bifunctional *hisNB* gene. All the Balneolota and *M. roseus* P3M-2 showed an N-terminal domain shorter than that of Bacteroidetes, which did not share any degree of sequence similarity with the HisN moiety of HisNB proteins. Moreover, a BLASTp search was performed to check whether this shorter domain could retrieve amino acid sequences of other known phosphatase: in the case of Balneolota, it retrieved only the Balneolota sequences, while in the case of *M. roseus* P3M-2, it retrieved only itself. Thus, whether this domain might perform the phosphatase activity is unclear.

The identification of a gene encoding a HOL-Pase in the genome of Chlorobi and Rhodothermota is much more complex. Experimental data demonstrated that the enzyme responsible for the sixth step of histidine biosynthesis may belong to (at least) two different families: (i) in *E. coli* and other enterobacteria, the HOL-Pase belongs to the DDDD superfamily, whereas (ii) in *Bacillus subtilis*, *Lactococcus lactis*, and *Saccharomyces cerevisiae*, the HOL-Pase belongs to the PHP family [41,42]. These two types of HOL-Pases do not share a significant degree of sequence similarity. It has also been shown that *E. coli hisN* is paralogous to *gmhB*, which codes for an enzyme involved in the dephosphorylation of D,D-heptose 1,7-PP, a precursor of the inner core of the outer membrane lipopolysaccharides [41,43]. In the β-proteobacterium *Neisseria meningitidis*, this protein is referred to as GmhX [44]. It has been suggested that *hisN* and *gmhB/gmhX* are the descendants of a common ancestral gene that encoded a DDDD-phosphatase with a broad substrate range and was able to catalyze at least the dephosphorylation of HOL-P and D,D-heptose 1,7-bisphosphate [41]. After the duplication event, the two copies diverged, narrowing their substrate specificity in such a way that one of them became a HOL-Pase and was recruited in histidine biosynthesis, whereas the other copy evolved towards a more specific GmhB protein. The common evolutionary origin of GmhB/GmhX and **γ**-proteobacterial HOL-Pase-coding genes led to the hypothesis that in some bacteria, the GmhB/GmhX enzyme might be a bifunctional protein being involved in both heptose and histidine biosynthesis, an idea also supported by phylogenetic analysis [41].

In order to identify a *hisN* candidate in the microorganisms where a bifunctional *hisNB* was not detected (i.e., the Chlorobi, Rhodothermota, and Balneolota), we probed all 91 members of the superphylum genomes using the amino acid sequence of each of the three phosphatases (*L. lactis* PHP type HOL-Pase, *E. coli* DDDD type HOL-Pase, and *E. coli* DDDD type GmhB) as a query. Data obtained for Chlorobi, Rhodothermota, and Balneolota are reported in Table 2 and revealed that:

i. The *L. lactis* PHP type sequence did not retrieve any sequence at an E-value below 1.0, suggesting the absence of such phosphatases in the considered groups.
ii. The *E. coli* DDDD HOL-Pase amino acid sequence (encoded by *hisN*) and GmhB amino acid sequence retrieved the same D,D-heptose 1,7-bisphosphate phosphatase from the genome of each bacterium belonging to Chlorobi. The *E. coli* GmhB retrieved a D,D-heptose 1,7-bisphosphate phosphatase for only two Balneolota out of four (*Aliifodinibius roseus* DSM 21986 and *Aliifodinibius sediminis* DSM 21194) and did not retrieve any sequence from Rhodothermota genomes, suggesting that bacteria belonging to these groups might use an enzyme belonging to a different family of phosphatases.

**Figure 1:**
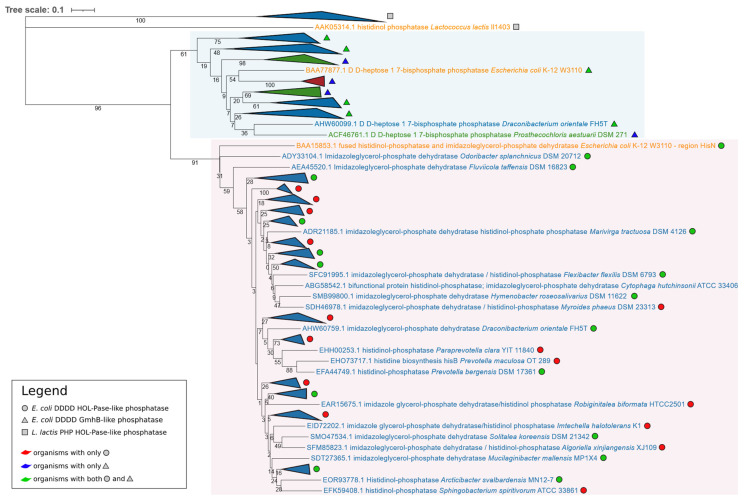
Phylogenetic tree based on the 125 phosphatase amino acid sequences retrieved from the BLASTp search using *L. lactis* PHP HOL-Pase, *E. coli* DDDD HOL-Pase, and *E. coli* DDDD GmhB as queries. After the multialignment trimming, 201 positions have been considered for the phylogenetic tree construction. Organisms belonging to the same taxonomic group and with the phosphatase belonging to the same family have been collapsed (the entire phylogenetic tree is available in Supplementary File S21, Figure S1). Colors light blue and pink identify the two main clusters. Proteins reported in orange are those from the tree queries, those in blue are from Bacteroidetes, those in green are from Chlorobi, and those in red are from Balneolota.

A total of 125 sequences were retrieved from the BLASTp search on the 91 genomes; the 125 sequences were aligned with ClustalW (Supplementary File S10) and the multialignment was used to construct the phylogenetic tree shown in Figure 1. Its analysis revealed that the sequences were split into two main different clusters separated by high bootstrap values (96) (highlighted in light blue and pink in Figure 1), the main of which (pink) included orthologs of the *E. coli* DDDD HOL-Pase, while the light blue cluster embedded orthologs of the *E. coli* functionally characterized GmhB amino acid sequence.

Concerning bacterial strains, the pink cluster was formed by only 72 sequences retrieved from the 72 Bacteroidetes, in full agreement with the presence in these bacteria of a bifunctional *hisNB* gene. Twenty-six of these organisms have 34 paralogs in the light blue cluster, a higher number than that theoretically expected (26); the higher number of paralogs is due to the fact that four and two of the Bacteroidetes possess two and three copies of GmhB amino acid sequences, respectively.

On the basis of the available data, we can depict the following evolutionary trajectories leading to the extant distribution of HOL-Pase-coding genes:

i. In some Bacteroidetes, a pair *gmhB*/*hisN* was found, suggesting that in these bacteria, the two genes are the descendants of an ancestral gene coding for a bifunctional enzyme catalyzing the sixth step of histidine biosynthesis and the heptose synthesis, in agreement with Jensen’s hypothesis [45].
ii. In Chlorobi, a single *gmhB* gene was found, and it can be speculated that it might code for a bifunctional enzyme, even though no experimental evidence in this sense is available.
iii. In other cases, the eighth step of histidine biosynthesis is catalyzed by a different DDDD HOL-Pase.

#### 3.2.3. Structure of *hisIE*

The *hisI* and *hisE* genes code for a phosphoribosyl-AMP cyclohydrolase and a phosphoribosyl-ATP phosphohydrolase, respectively, which are responsible for the third and second steps of histidine biosynthesis. In some microorganisms they are fused to form a bifunctional gene. The analysis of the phylogenetic distribution of *hisIE* bifunctional genes carried out previously [3] revealed that it is a paradigmatic example of divergent evolution, convergent evolution, and horizontal gene transfer. A *hisIE* bifunctional gene was found in the majority of the Bacteroidetes, Rhodothermota, Balneolota, and Chlorobi genomes analyzed; indeed, only 6 strains out of 91 (belonging to the Rhodothermota, Balneolota, and Bacteroidetes phyla) harbor the *hisI* and *hisE* genes spatially separated along the chromosome (Multialignment, Supplementary File S5).

However, in Chlorobi (except for *M. roseus* P3M-2 and *I. album* JCM 16511) and Rhodothermota (except for those organisms having two separated genes), the *hisIE* gene is much shorter than the “canonical” *E. coli*-type bifunctional gene. Indeed, the encoded proteins are about 65 residues shorter than those encoded by the Bacteroidetes orthologs, for a total length of about 137 amino acids. Interestingly, the analysis of the multialignment revealed that the Chlorobi-Rhodothermota *hisIE* gene harbors the entire 5′ moiety (corresponding to the *hisI* counterpart); on the other hand, the *hisE* moiety is “minimized” to a short region of about 19–25 residues, which is highly conserved between the Chlorobi-Rhodothermota HisIE sequences and easily alignable to the region spanning from residue 146 to amino acid 168 of the Bacteroidetes orthologs. From the alignment of Chlorobi HisIE sequences, it was possible to notice a high conservation of amino acid residues along the entire sequence, except for a region comprised of residues 113–118; this region could be a potential “linker” region between HisI and HisE moieties without a specific function, except that of connecting the two catalytic domains of the enzyme, and thus less conserved during evolution.

To the best of our knowledge, this is the first time that such a structure has been identified. It is quite possible that this short region might be able to perform the reaction catalyzed by the phosphoribosyl-ATP pyrophosphatase encoded by *hisE*. It is known that the enzymatic steps of histidine biosynthesis are the same in all histidine-synthesizing organisms. Hence, even though there is no experimental evidence of such hypothesis, the finding that no other sequences with a significant degree of sequence similarity with HisE were disclosed in the genome of Chlorobi supports this idea. If this “minimized” version of the HisIE protein was able to perform both the second and third steps of histidine biosynthesis, this would raise the intriguing question of why this event occurred only in Chlorobi.

#### 3.2.4. Structure of *hisG* and *hisZ*

It has been reported that *hisG*, the gene coding for the first enzyme of histidine biosynthesis (ATP-phosphoribosyltransferase, ATP-PRT), exists in two alternative forms, a long version which is called *hisG_L_*, corresponding to the *E. coli* gene, and a shorter one, referred to as *hisG_S_*, which was first characterized in *L. lactis* [15]. The main difference between the two HisG types concerns their length: 292 (±11) and 214 (±9) amino acids for HisG_L_ and HisG_S_, respectively (Fondi, unpublished results), a difference ascribed to the lack of a C-terminal domain involved in the feedback inhibition of the enzyme in HisG_S_. In those (micro)organisms harboring *hisG_S_*, the missing regulatory region is replaced by another gene, *hisZ*, whose product is able to bind to HisG_S_ and regulate its activity [15]. Therefore, it can be assumed that all histidine-producing organisms harboring *hisG_L_* should lack *hisZ*, and vice versa, those possessing *hisG_S_* should harbor *hisZ*.

Furthermore, *hisZ* shares a high degree of sequence similarity with the gene encoding histidyl-tRNA synthetase (HisRS). HisZ proteins are shorter than HisRSs, lacking a C-terminal region, which represents the site of HisRS binding to tRNA^His^. The average length is 377 (±39) residues for the former and 445 (±38) for the latter (Fondi, unpublished results).

A deep analysis of the phylogenetic distribution of *hisG_L_*, *hisG_S_*, and *hisZ* has not been carried out up to now; thus, we checked the presence of these genes in the Bacteroidetes, Chlorobi, Rhodothermota, and Balneolota genomes. From the search on UniProtKB for HisG, all the organisms resulted in harboring only the *hisG* gene; probing with the *L. lactis* HisZ sequence did not retrieve any sequence from any of the 91 genomes. These data suggested that the *hisZ* gene was absent from each of these genomes. This, in turn, would imply that the HisG enzyme from these bacteria should belong to the HisG_L_ type. To check this hypothesis, all 91 HisG proteins were aligned as described in the Materials and Methods. According to these data, all 91 sequences belong to HisG_L_ proteins (Multialignment, Supplementary File S6).

Moreover, Chlorobi (except for *M. roseus* P3M-2 and *I. album* JCM 16511) and three Rhodothermota (*Rubrivirga marina* SAORIC-28, *Rhodothermus profundi* DSM 22212, and *Rhodothermus marinus* DSM 4252) out of six harbor a different HisG sequence compared to the other organisms, with an additional region of five residues in the position between amino acids 93 and 98 and a sequence similarity with that of the others of less than 51%.

#### 3.2.5. Structure of *hisD*, *hisC*, and *hisH*

The *hisD*, *hisC*, and *hisH* histidine biosynthetic genes code for a histidinol dehydrogenase, a histidinol-phosphate aminotransferase, and the glutamine amidotransferase subunit of the IGPS, respectively. Their gene products are respectively responsible for the ninth and tenth steps (HisD), the seventh step (HisC) and the fifth step (HisH-HisF complex) of the histidine biosynthetic pathway. The analysis of their amino acid sequence in the organisms considered in the present work revealed that HisD and HisH are conserved among the members of the superphylum, with no specific structures for the different taxonomic groups. The multialignment of the HisC amino acid sequences revealed that Chlorobi (except for *I. album* JCM 16511 and *M. roseus* P3M-2) and Rhodothermota have an additional region of about seven residues at the *N*-terminal of the protein (Multialignment, Supplementary Files S7–S9). This aspect, together with the sharing of the same additional HisA *C*-terminal region among the same organisms, suggests either a possible HGT of histidine biosynthetic genes between these two groups or a molecular rearrangement that occurred in the ancestor of the two groups.

#### 3.2.6. Organization of Histidine Biosynthetic Genes

In principle, and according to Fondi et al. [2], we can depict the following possible scenarios for the organization of genes involved in the same metabolic pathway:

i. Genes organized in *homogeneous* (and more or less compact) *operons*; in this case, the operon embeds only genes involved in the same metabolic pathway.
ii. Genes organized in *heterogeneous* (and more or less compact) *operons*; such operons include genes involved in the same metabolic pathways and one or more “alien” genes (genes apparently not involved in the same metabolic route and having homologs in other species) [2] or “ORFan” genes (lacking homologs in closely related species and probably acquired from bacteriophages) [46] responsible for other metabolic abilities.
iii. Genes organized in *homogeneous* and/or *heterogeneous* (and more or less compact) *suboperons*.
iv. Genes partially scattered and partially organized in *homogeneous* and/or *heterogeneous suboperons*.
v. Genes completely scattered (regulons).

**Figure 2:**
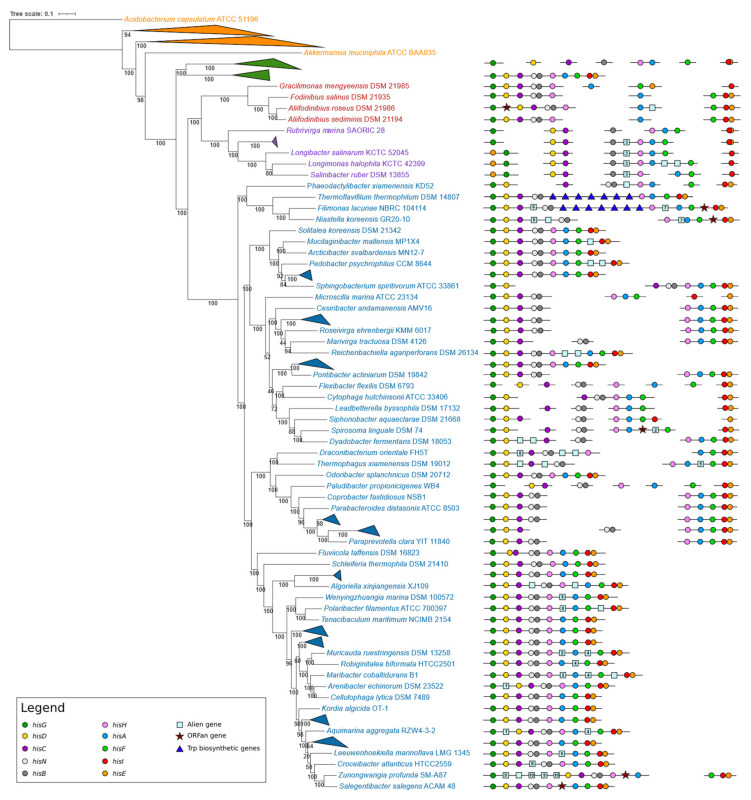
Phylogenetic tree based on the concatenation of 182 conserved proteins of the 91 superphylum organisms and 17 outgroups. After the multialignment trimming, 47,919 positions have been considered for the phylogenetic tree construction. Organisms reported in orange are the outgroups, those in blue are Bacteroidetes, those in green are Chlorobi, those in red are Balneolota, and those in violet are Rhodothermota. Genomic organization of *his* genes is represented on the right side of the figure. The outgroups and those organisms belonging to the same taxonomic group sharing the same genomic organization of *his* genes have been collapsed (the entire phylogenetic tree is available in Supplementary File S21, Figure S2). Numbers within the alien gene icons represent groups of proteins with a significant BLASTp E-value (Table S3, Supplementary File S1). Information about organisms’ growth temperature range is reported next to organisms’ names in the entire phylogenetic tree available in Supplementary File S21, Figure S2.

The organization of *his* biosynthetic genes in the four different groups is reported in Figure 2, whose analysis revealed a large variety of *his* genes organizations.

*Chlorobi.* The analysis of the organization of histidine biosynthetic genes from the nine Chlorobi revealed that in seven of them, the histidine biosynthetic genes are scattered throughout the genome, with the only exception of *hisH* and *hisA*, which in most cases are separated by a short intergenic region, suggesting that they might be organized in a bicistronic operon. In two cases (*I. album* JCM 16511 and *M. roseus* P3M-2), the *his* genes are arranged in a compact operon with a bifunctional *hisNB* gene and a bifunctional *hisIE* gene larger than that found in the other members of the same group. It is quite possible that the entire *his* operon of these two Chlorobiota may be acquired via HGT. This is also suggested by the analysis of the phylogenetic trees based on the amino acid sequence of His proteins (see Supplementary File S21) where the *I. album* JCM 16511 and *M. roseus* P3M-2 sequences clustered with strains whose *his* genes are arranged in operons. This is also in agreement with the presence and structure of bifunctional *hisNB* and *hisIE* genes in the two operons.

*Rhodothermota and Balneolota.* Members of these groups showed a partial clustering of *his* biosynthetic genes, with some genes scattered and others clustered in suboperons (of different length), also including some alien genes (reported in Table S3, Supplementary File S1). With the only exceptions being *Salinibacter ruber* DSM 13855, *Longimonas halophila* KCTC 42399, and *Longibacter salinarum* KCTC 52045, where the *his* genes are arranged in mini-operons and *hisE* is located upstream *hisG*, they maintain the same gene relative order. It is also quite possible that (at least some genes) might have undergone HGT events, as shown by the phylogenetic trees constructed using the amino acid sequences of His proteins (see for instance the HisD, HisA, HisC, and HisG trees shown in Supplementary File S21). No compact entire, either heterogeneous or homogeneous, operons have been found in these groups.

*Bacteroidetes.* A large variety of organization strategies for *his* genes have been disclosed in the genome of Bacteroidetes. The analysis of Figure 2 witnesses a progressive clustering of *his* genes. In many cases, the *his* genes are arranged in compact homogenous operons. In other cases, alien and ORFan genes are still present between some of the *his* genes (reported in Table S3, Supplementary File S1). These ORFan or alien genes are located mainly in larger operons rather than in suboperons. This might suggest that these genes may be the remnant of recombination events that might have joined suboperons. In some cases, the same alien gene is located in the same position in *his* (sub)operons of different microorganisms; as an example, the alien gene 3 is located between *hisB* and *hisH* genes in all the Rhodothermota (with the only exception of *Rubrivirga marina* SAORIC-28). Other examples can be seen in the right side of Figure 2.

When the nine *his* biosynthetic genes are arranged in a single (putative) operon, their relative order (*hisGDCNBHAFIE*) is the same as that of the **γ**-proteobacterial orthologous operons. In some genomes, these operons also contain genes apparently not involved in the histidine biosynthesis. Interestingly, in *Thermoflavifilum thermophilum* DSM 14807 and *Filimonas lacunae* NBRC 104114, the histidine biosynthetic operon has been interrupted by the introgression of tryptophan biosynthetic genes. Even though no data concerning the expression of *his* and *trp* genes is available for these microorganisms, the intergenic regions existing between the different genes of this unusual cluster are very short, suggesting that they might belong to the same transcriptional unit, i.e., the same operon. In some Bacteroidetes, the histidine biosynthetic genes are split into two shorter operons (suboperons) of different lengths and containing a different number of *his* genes; however, in each of these suboperons, the *his* genes maintain the same relative order of the longer ones. The genome of other Bacteroidetes contains *his* genes showing a more complex organization, with genes scattered and organized in small operons to a different extent in different organisms, without an apparent scheme, except for the fact that, independently from the length and the number of the suboperons, the relative order of the *his* genes embedded in the suboperons is the same in all cases.

#### 3.2.7. “Unusual” *his* Gene Structures and Organization

*M. roseus* P3M-2 (Chlorobiota), *Chryseobacterium gleum* NCTC11432 (Bacteroidota), and *Maribacter cobaltidurans* B1 (Bacteroidota) harbor an additional copy of *hisF* and *hisH* genes, referred to, respectively, as *hisF2* and *hisH2*, not located in the histidine operon. In *C. gleum* NCTC11432, the second copies of these two genes are separated by just seven nucleotides, suggesting that they could belong to the same transcriptional unit, thus forming a bicistronic operon. It is known that in Eukarya, the two genes are fused to form a bifunctional gene (HIS7) encoding an IGPS [3,41]. It has been proposed that HIS7 is the outcome of a fusion event involving two distinct but very close cistrons. Since all histidine-synthesizing Eukarya harbor a bifunctional HIS7 gene, it is quite possible that either the fusion event occurred in the common ancestor of all Eukarya, or an already formed bifunctional gene was horizontally transferred to Eukarya from a (still unknown) prokaryote. In order to check the latter hypothesis, a phylogenetic tree constructed using the concatenated sequences of HisH and HisF (and HisH2 and HisF2) of some representatives of Bacteria, Archaea, and Eukarya was constructed, whose analysis revealed that the HisH2 and HisF2 amino acid sequences do not join the eukaryal sequences, thus excluding the possibility that the bicistronic operon *hisH2*-*hisF2* might have been donated to eukaryotes (see Supplementary File S21, Figure S11).

### 3.3. Phylogenetic Analyses

In order to assess the existence of a correlation between the taxonomical position and the structure and organization of *his* genes, their structure/organization was mapped onto a phylogenetic tree that was constructed on a set of orthologous genes retrieved using the BBH approach (as described in Materials and Methods). The pairwise bidirectional search was performed between every gene present in *Bacteroides fragilis* NCTC 9343 (4346 amino acidic sequences, for a total of 68,390 blastp searches) and the proteomes of each of the 108 organisms (91 ingroups and 17 outgroups, listed in Tables S1 and S2, Supplementary File S1) under investigation. The BBH algorithm automatically discarded 4164 proteins whose function was not conserved across all samples, i.e., those for which the best hit was not bidirectional, leaving a total of 182 suitable orthologs. Subsequently, with the intent of having a wider prospective on the clusters of orthologous group (COG) categories that were allowed in by the BBH selection, all 182 were uploaded to eggNOG-mapper webserver (as described in Materials and Methods). The most populated category was J—Translation, ribosomal structure, and biogenesis, having 69/182 genes; followed by L—Replication, recombination, and repair, with 20/182 genes represented; F—Nucleotide transport and metabolism (16/182); H—Coenzyme transport and metabolism—(15/182); and O—Post-translational modification, protein turnover, chaperones (13/182). All the remaining categories were represented by less than 10 orthologs (Table S4, Supplementary File S1). The MSA of the 182 selected orthologs was performed with MAFFT and concatenated to have an initial number of 113,290 amino acidic positions for each of the 108 organisms. After filtering, each sequence in the final alignment was composed of 47,919 positions (Supplementary File S11) and the concatenamer was forwarded to MEGA7 for phylogenetic analysis (as described in Materials and Methods).

The analysis of the phylogenetic tree revealed that a progressive clustering of *his* genes occurred starting from Chlorobi up to Bacteroidetes (Figure 2). However, some discrepancies between taxonomy and gene organization can be observed. For instance, the *I. album* JCM 16511 and *M. roseus* P3M-2 compact operons are unlike the scattered *his* genes in the other Chlorobi. This discrepancy can be explained on the basis of possible HGT events from members of other groups to these two bacteria.

According to this idea, in the phylogenetic trees (Supplementary Files S12–S21) constructed using the amino acid sequence of His proteins of the 91 members of the superphylum and of the 17 outgroups, these two Chlorobi are placed distantly from the other members of the phylum and generally intermixed with members of Bacteroidetes. The (apparent) contradictory correlation between the taxonomical position of *I. album* JCM 16511 and *M. roseus* P3M-2 and the operonic organization of their *his* biosynthetic genes might be explained on the basis of a horizontal transfer of the entire operon involving members of Bacteroidota and/or Balneolota. Moreover, the analysis of the phylogenetic trees constructed using the amino acid sequence of single His proteins revealed the existence of horizontal gene transfer events between different members of this bacterial superphylum, also belonging to different genera/species, as well as between members of taxonomically distant groups.

The possibility of extensive HGT events involving one or more *his* genes or the complete operon in this group is in agreement with the idea that the *his* operon is an open *plastic* operon, i.e., an operon in which different gene organizations are possible [4,11]. In fact, this idea can be reformulated as follows: “the structure and organization of histidine biosynthetic genes is plastic”, i.e., different structural and organization arrays are possible.

### 3.4. A Model for the Evolution of Histidine Biosynthetic Genes Structure and Organization in Bacteroidetes/Chlorobi/Balneolota/Rhodothermota

In principle, two different and mutually exclusive scenarios can be depicted to explain the great variety of organization strategies of the *his* genes in this superphylum. The first one predicts that in the last common ancestor (LCA) of this group, the *his* genes were organized in a compact homogeneous operon. According to this scenario, in the different phylogenetic lineages, the operon would have undergone different and more or less large molecular rearrangements, separating the *his* genes either into suboperons and/or regulons. The second one predicts, with regard to the proteobacterial *his* genes, that the LCA of this group possessed scattered *his* genes, which, over time, should have clustered together via a piecewise mechanism [12], firstly building mini-operons and then joining them, giving rise—in the end—to homogenous compact *his* operons.

We cannot exclude a priori the possibility that the *his* genes were “operonically” organized in the LCA of this group. However, we can speculate that the heterogeneous distribution and organization of *his* genes in this bacterial group suggest the absence of a complete histidine biosynthetic operon in their common ancestor. Indeed, the presence of a completely formed compact *his* operon resembling the *E. coli* one very early in the evolution of this bacterial group does not appear to be supported by the comparative analysis of *his* genes and their structure and organization. If the idea of the antiquity of the *his* operon was correct, then a high number of (independent) molecular rearrangements (including formation of new and very similar, if not identical, promoter sequences upstream of each separated gene and the destroying of genes encoding bifunctional enzymes) would be necessary to explain the extant scenario (see also [13]). Based on this assumption, we suggest that the assembly of compact *his* operons may have occurred via the progressive clustering of pre-existing suboperons embedding part of the genes of the final and completely assembled compact operon. This is in agreement with the ‘‘piecewise’’ model proposed by Fani et al. [12,13] to explain the mechanisms responsible for the building up of complex operons.

The analysis of the phylogenetic trees constructed using the single His proteins revealed an extensive lateral transfer. These events occurred not only between members of the bacterial group analyzed in this work but also between strains of this group and phylogenetically distant bacteria. This finding is also in agreement with data reported previously for Archaeal [9] and proteobacterial [12,13] *his* genes. The lateral transfer of genes between taxonomically distant microorganisms that might have different transcriptional signals for the expression of such genes in the recipient strains implies that the barriers to heterologous gene expression might be overcome by modifying, in a short timescale, the regulatory signals of the introgressed genes, as shown by Dabizzi et al. [47].

A remaining question concerns the force(s) that might have led to the compacting of genes to form operons. As previously reported ([2] and references therein), different hypotheses have been proposed to explain the origin of operons. According to the different models, different forces, not mutually exclusive, might have driven the compacting of genes into operons, such as the necessity of equimolarity and/or coregulation or the formation of metabolon-like structures.

It is quite interesting that, in almost all genomes analyzed, one of the most shared mini-operons contains the three genes *hisH*, *hisA*, and *hisF* that code for enzymes catalyzing the two steps involved in the cross connection of histidine biosynthesis, nitrogen metabolism, and the de novo synthesis of purines. As reported in the Introduction, it is known that HisH and HisF interact both in vitro and in vivo to form the bifunctional enzyme IGPS [48]. Thus, the possibility that the three proteins HisA, HisH, and HisF might act in a concerted manner forming a metabolon-like complex, which, in turn, should require the three proteins to be colocalized in the same cellular microenvironment, cannot be excluded a priori. This might be assured/favored by an operonic organization of the three coding genes. This idea is also in agreement with the finding that in those Chlorobi harboring scattered *his* genes, *hisH* and *hisA* are also organized in a bicistronic operon. The formation of a supramolecular structure fits the Glansdorff hypothesis on the origin of operons; in his paper, Glansdorff [49] suggested that a key role in the assembly of genes into operons was covered by the adaptation to thermophily of the early cells. The transcription-translation coupling occurring in prokaryotes supports this idea (Ref. [12] and references therein). To check for the existence of a possible correlation between the Bacteroidetes/Chlorobiota/Balneolota/Rhodothermota lifestyle and the organization of their histidine biosynthetic genes, as well as the most probable operon formation driving forces, we reported the thermophily/hyperthermophily of the corresponding taxa in the two phylogenetic trees shown in Figure 2. Our results revealed that apparently no correlation exists between thermophily and the *his* gene organization. Indeed, thermophilic bacteria exhibit a high variety of *his* gene organization. This is also true for mesophilic strains. However, the analysis of the phylogenetic tree reported in Figure 2 revealed that all the cold-adapted microorganisms harbor *his* biosynthetic genes organized in compact and in some cases homogeneous operons, independently from the organization of the *his* genes in the surrounding microorganisms of the same branch. Even though the number of cold-adapted microorganisms belonging to this group is limited, it is possible that adaptation to low temperature might have played a role in the organization of histidine biosynthetic genes, an issue which deserves further analyses. In addition to the formation of metabolon-like structures, it is possible that other different forces might have driven the assembly of more or less compact *his* operons in this bacterial group; these at least include the necessity of equimolarity and/or coregulation.

## 4. Conclusions

The analysis of the large variety of *his* genes structures and organizations observed in this bacterial group revealed completely different scenarios with genes organized in more or less compact, heterogeneous (embedding alien and/or ORFan genes), or homogeneous operons, in sub-operons, or in regulons.

The organization array of *his* genes in the extant Bacteroidetes/Chlorobiota/Balneolota/Rhodothermota lineages speaks toward a piecewise construction of *his* suboperons [12,13], suggesting that in the LCA of this group, *his* genes were scattered throughout the chromosome and that different forces have driven the assembly of *his* genes in compact operons.

Gene fusion events (i.e., *hisIE* and *hisNB*) and/or paralog formation (i.e., the pair *hisA*/*hisF*, the multiple copies of *hisH* and *hisF*, and the gene pair *hisN* and *gmhB*), as well as other molecular rearrangements not yet identified (the *hisIE* “minimization” in the genome of Chlorobi), have shaped the *his* gene structure in this group. In addition to this, the analysis of the amino acid sequence of HisA from some strains revealed the existence of a degree of sequence similarity between the eight modules, sufficiently high enough to suggest that this gene is the result of a cascade of elongation events from an ancestral module an eighth of the size of the extant gene, gesturing towards a divergent evolution of TIM-barrel coding genes.

Moreover, HGT of single genes or entire operons might have been the basis of genes exchanging between strains of the same group, between strains of different groups, and between other bacteria.

## Figures and Tables

**Table 1:** Degree of sequence identity (upper half of the table) and similarity (lower half) between the eight HisA modules of *D. fermentas* DSM 18053. Numbers: 1, 2, 3, 4, 5, 6, 7, 8 correspond to aa 1–30, 31–60, 61–90, 91–120, 121–150, 151–180, 181–210, 211–240 of the HisA sequence, respectively.

Module	1	2	3	4	5	6	7	8
1	-	16.6	9.3	13.3	18.7	6.0	14.7	9.6
2	43.3	-	18.1	13.3	9.6	15.1	9.0	15.6
3	40.5	36.3	-	10.0	9.3	11.7	15.1	6.0
4	40.0	30.0	26.6	-	16.6	12.1	6.2	14.3
5	40.6	35.4	34.3	36.1	-	18.1	13.3	16.6
6	36.0	39.4	32.3	27.2	39.4	-	13.3	16.1
7	44.1	45.4	45.4	37.5	40.0	40.0	-	15.1
8	45.1	40.6	43.3	34.3	47.2	38.7	42.4	-

**Table 2:** *L. lactis* PHP HOL-Pase, *E. coli* DDDD HOL-Pase, and *E. coli* DDDD GmhB were used as queries to search for an HisN candidate in Chlorobi, Balneolota, and Rhodothermota genomes using BLASTp. For those organisms for which a phosphatase was found, the NCBI accession number of the enzyme is reported.

Phylum	Organism	*L. lactis* PHP HOL-Pase	*E. coli* DDDD HOL-Pase	*E. coli* DDDD GmhB
Chlorobi	*Chlorobaculum limnaeum* DSM 1677	-	AOS84578.1	AOS84578.1
	*Chlorobaculum tepidum* TLS	-	AAM73229.1	AAM73229.1
	*Chlorobium limicola* DSM 245	-	ACD91212.1	ACD91212.1
	*Chlorobium luteolum* DSM 273	-	ABB23109.1	ABB23109.1
	*Chlorobium phaeobacteroides* DSM 266	-	ABL66364.1	ABL66364.1
	*Chloroherpeton thalassium* ATCC 35110	-	ACF14131.1	ACF14131.1
	*Ignavibacterium album* JCM 16511	-	AFH49163.1	AFH49163.1
	*Melioribacter roseus* P3M-2	-	AFN73296.1	AFN73296.1
	*Prosthecochloris aestuarii* DSM 271	-	ACF46761.1	ACF46761.1 ACF47029.1
Balneolota	*Aliifodinibius roseus* DSM 21986	-	-	SHF20111.1
	*Aliifodinibius sediminis* DSM 21194	-	-	SMO32123.1
	*Fodinibius salinus* DSM 21935	-	-	-
	*Gracilimonas mengyeensis* DSM 21985	-	-	-
Rhodothermota	*Longibacter salinarum* KCTC 52045	-	-	-
	*Longimonas halophila* KCTC 42399	-	-	-
	*Rhodothermus marinus* DSM 4252	-	-	-
	*Rhodothermus profundi* DSM 22212	-	-	-
	*Rubrivirga marina* SAORIC-28	-	-	-
	*Salinibacter ruber* DSM 13855	-	-	-

## Data Availability

Accession numbers of sequences used in this work are reported in Supplementary File S1.

## Supplementary Materials

The following are available online at https://www.mdpi.com/article/10.3390/microorganisms9071439/s1. Supplementary File S1: file containing a table of the organisms of the superphylum considered in the present work (Table S1), a table of the outgroups (Table S2), a table of the alien and ORFan genes (Table S3), and a list of the 182 conserved proteins (Table S4); Supplementary Files S2–S9: multialignments of the histidine protein amino acid sequences of the 91 ingroups in FASTA format. Supplementary Files S10–S20: trimmed multi-alignments used for the construction of the phylogenetic trees in FASTA format; Supplementary File S21: phylogenetic trees.
