# Supplementary material for: The Histidine Biosynthetic Genes in the Superphylum Bacteroidota-Rhodothermota-Balneolota-Chlorobiota: Insights into the Evolution of Gene Structure and Organization
Source: Microorganisms. 2021 Jul 3;9(7):1439. doi: 10.3390/microorganisms9071439 (PMC8305728; doi:10.3390/microorganisms9071439)
Supplement: Supplementary file 1 [file microorganisms-09-01439-s001.zip › Supplementary file S21.pdf]

The histidine biosynthetic genes in the superphylum Bacteroidota-Rhodothermota-Balneolota-Chlorobiota: insights into the evolution of gene structure and organization

Supplementary Material – Phylogenetic trees

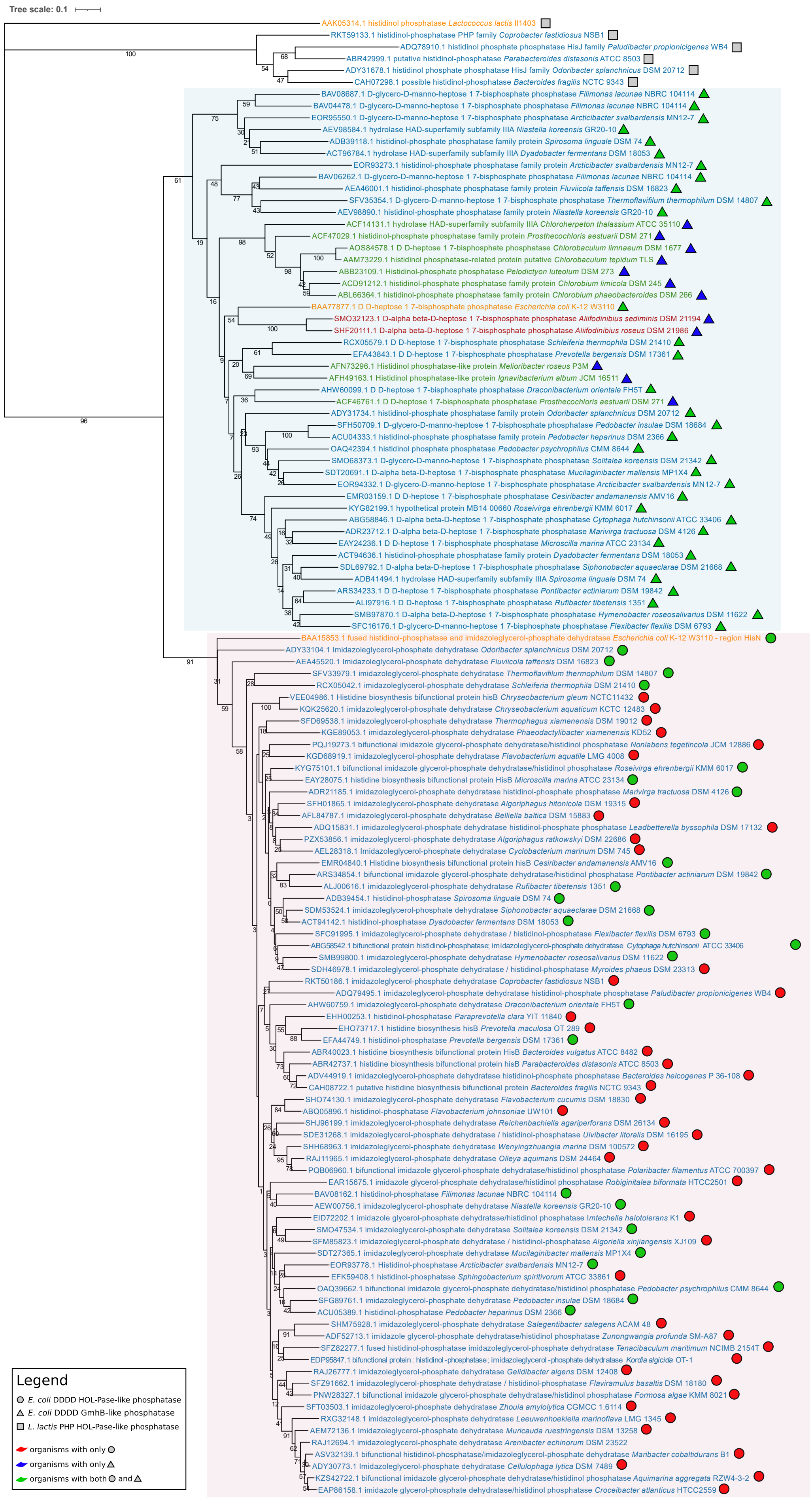

Figure S1. Phylogenetic tree based on the 125 phosphatases amino acid sequences retrieved from the BLASTp search using *L. lactis* PHP HOL-Pase, *E. coli* DDDD HOL-Pase, and *E. coli* DDDD GmhB as queries. After the multi-alignment trimming, 201 positions have been considered for the phylogenetic tree construction. Colors light blue and pink identify the two main clusters. Proteins reported in orange are those from the tree queries, those in blue are from Bacteroidetes, in green from Chlorobi, and in red from Balneolota.

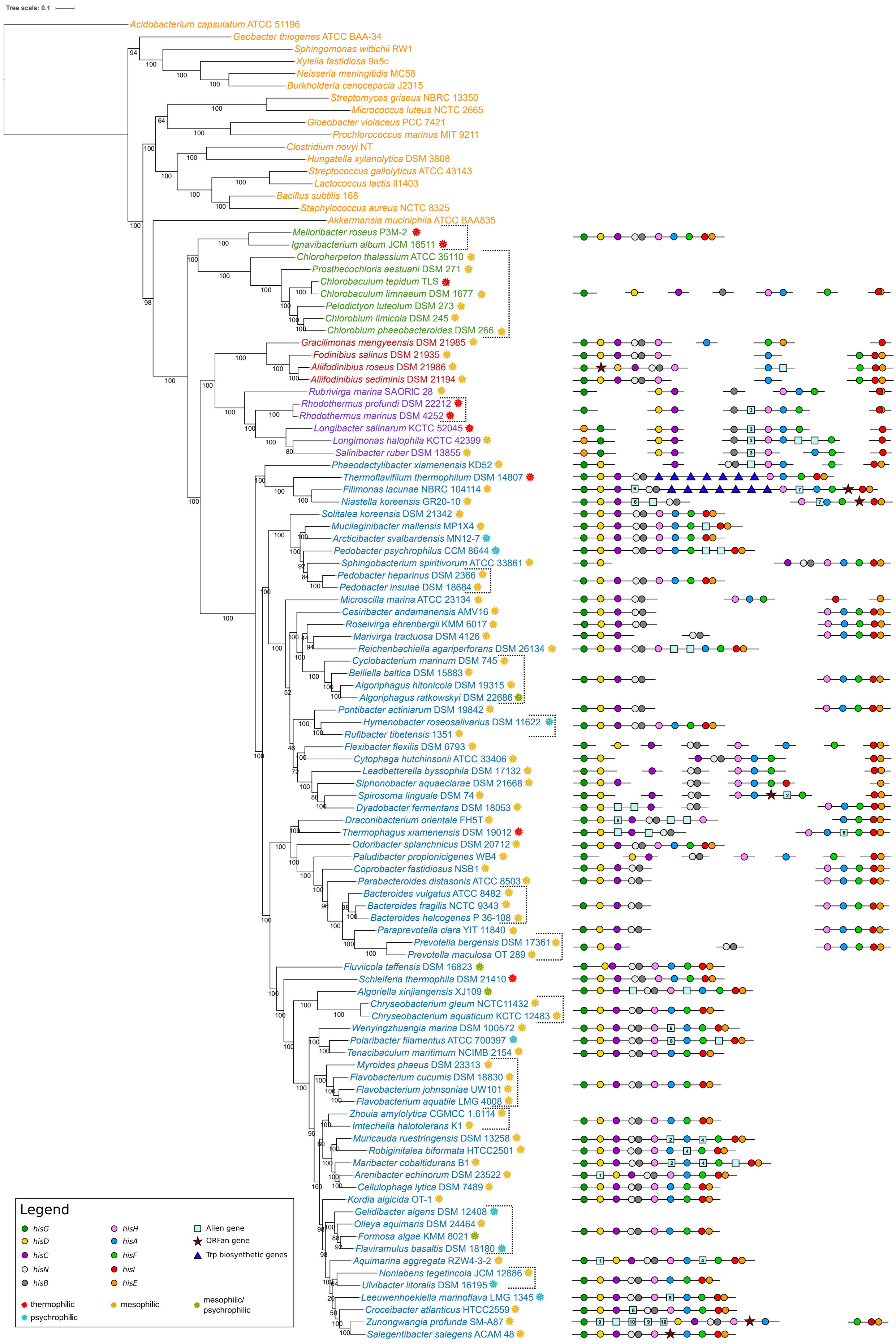

Figure S2. Phylogenetic tree based on the concatenation of 182 conserved proteins of the 91 superphylum organisms and 17 outgroups. After the multi-alignment trimming, 47919 positions have been considered for the phylogenetic tree construction. Organisms reported in orange are the outgroups, those in blue are Bacteroidetes, in green Chlorobi, in red Balneolota, and in violet Rhodothermota. Genomic organization of *his* genes is represented on the right side of the figure. Numbers within the alien gene icons represent groups of proteins with a significant BLASTp E-value (Table S3, Additional file S1). Information about organisms growth temperature range is reported next to organisms names.

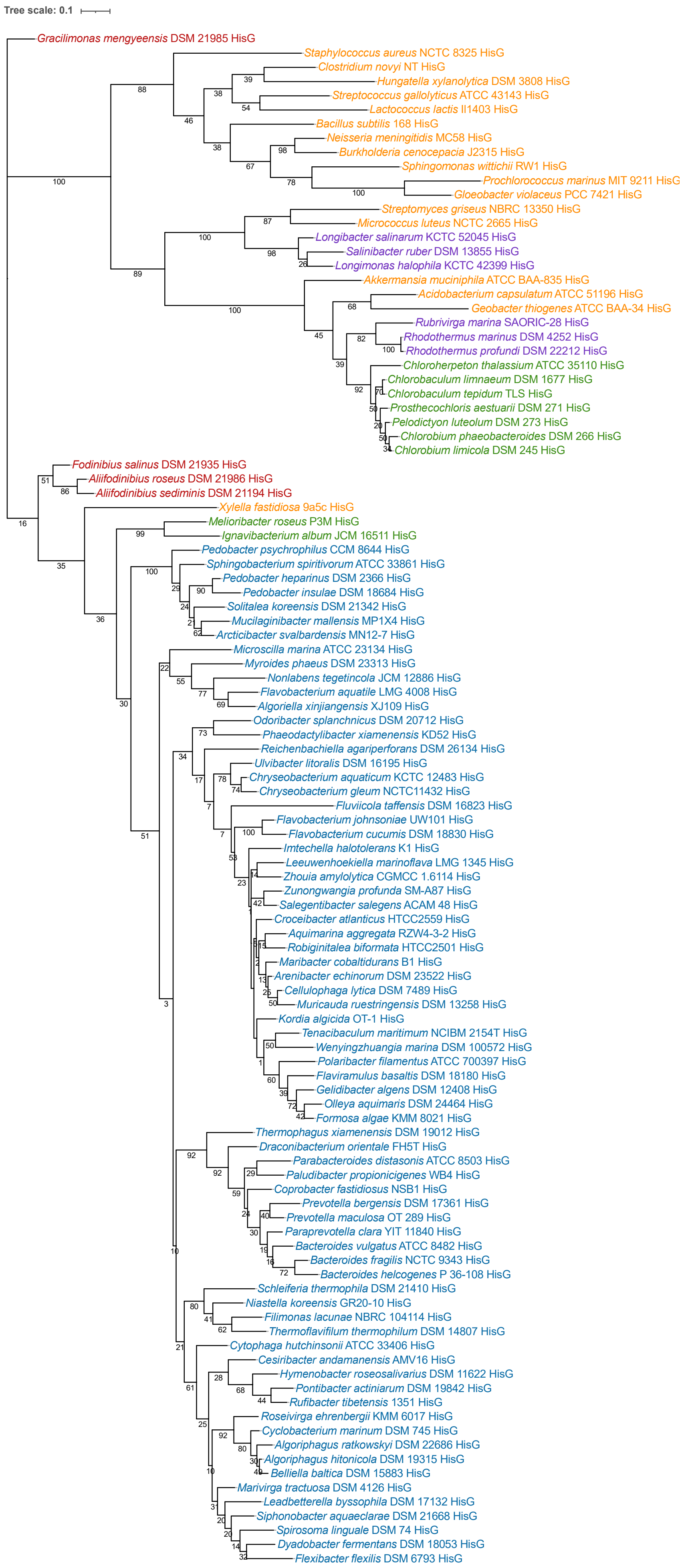

Figure S3. Phylogenetic tree based on the HisG amino acid sequences of the 91 superphylum organisms and 17 outgroups. After the multi-alignment trimming, 249 positions have been considered for the phylogenetic tree construction. Organisms reported in orange are the outgroups. Those in blue are Bacteroidetes, in green Chlorobi, in red Balneolota, and in violet Rhodothermota.

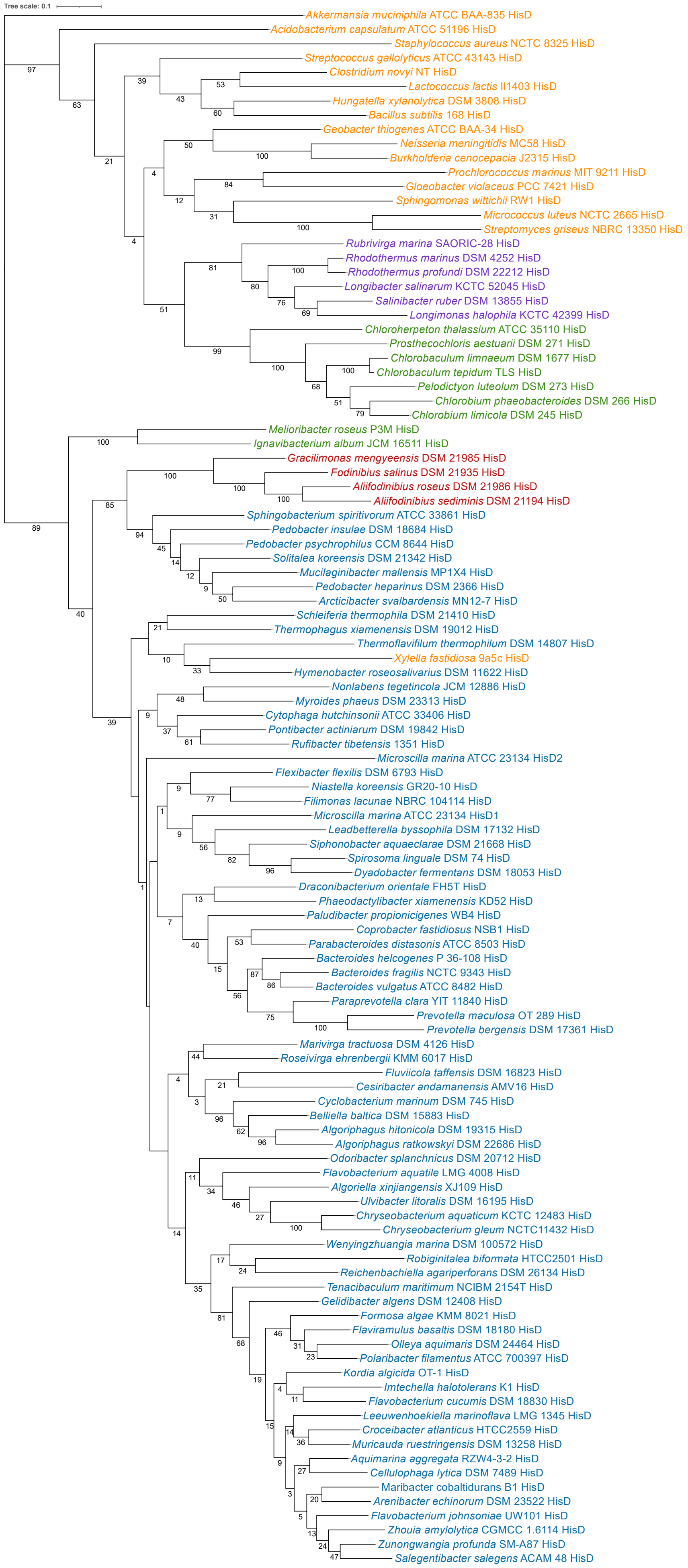

Figure S4. Phylogenetic tree based on the HisD amino acid sequences of the 91 superphylum organisms and 17 outgroups. After the multi-alignment trimming, 372 positions have been considered for the phylogenetic tree construction. Organisms reported in orange are the outgroups. Those in blue are Bacteroidetes, in green Chlorobi, in red Balneolota, and in violet Rhodothermota.

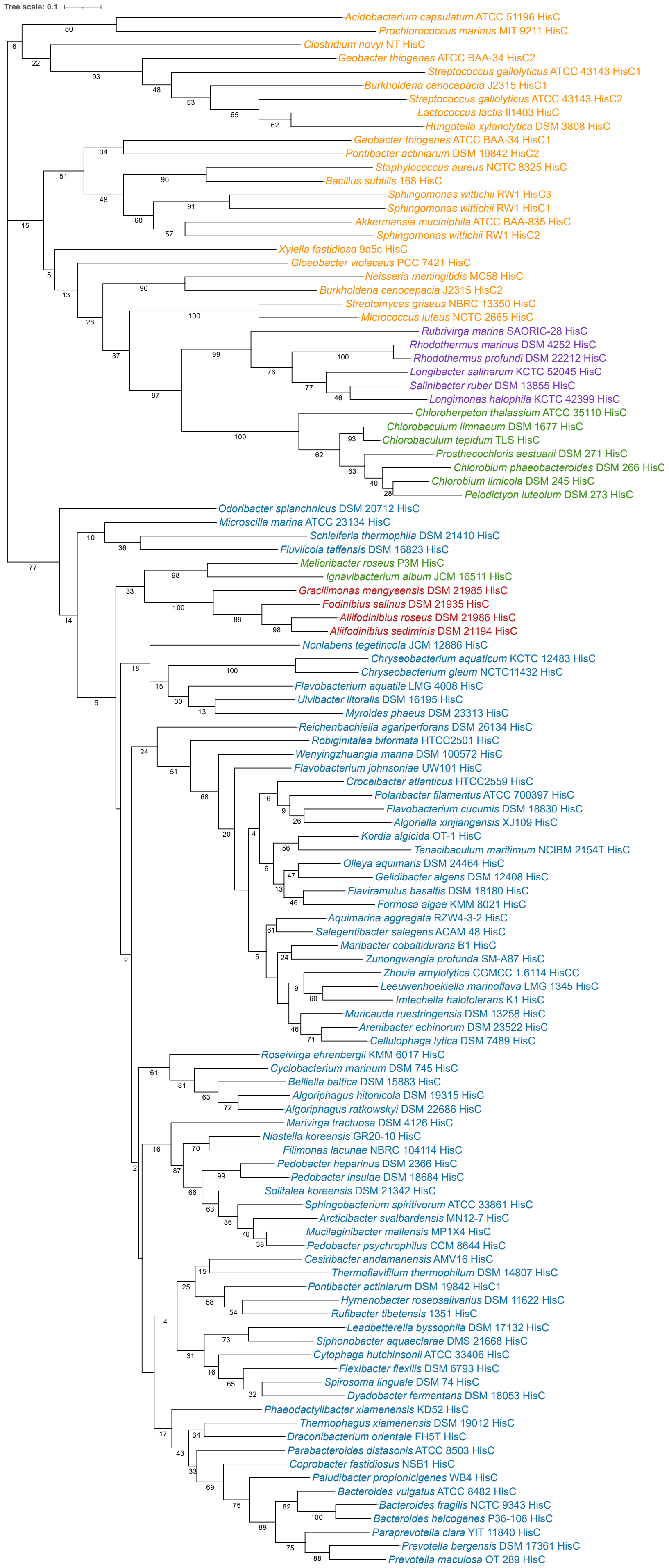

Figure S5. Phylogenetic tree based on the HisC amino acid sequences of the 91 superphylum organisms and 17 outgroups. After the multi-alignment trimming, 301 positions have been considered for the phylogenetic tree construction. Organisms reported in orange are the outgroups. Those in blue are Bacteroidetes, in green Chlorobi, in red Balneolota, and in violet Rhodothermota.

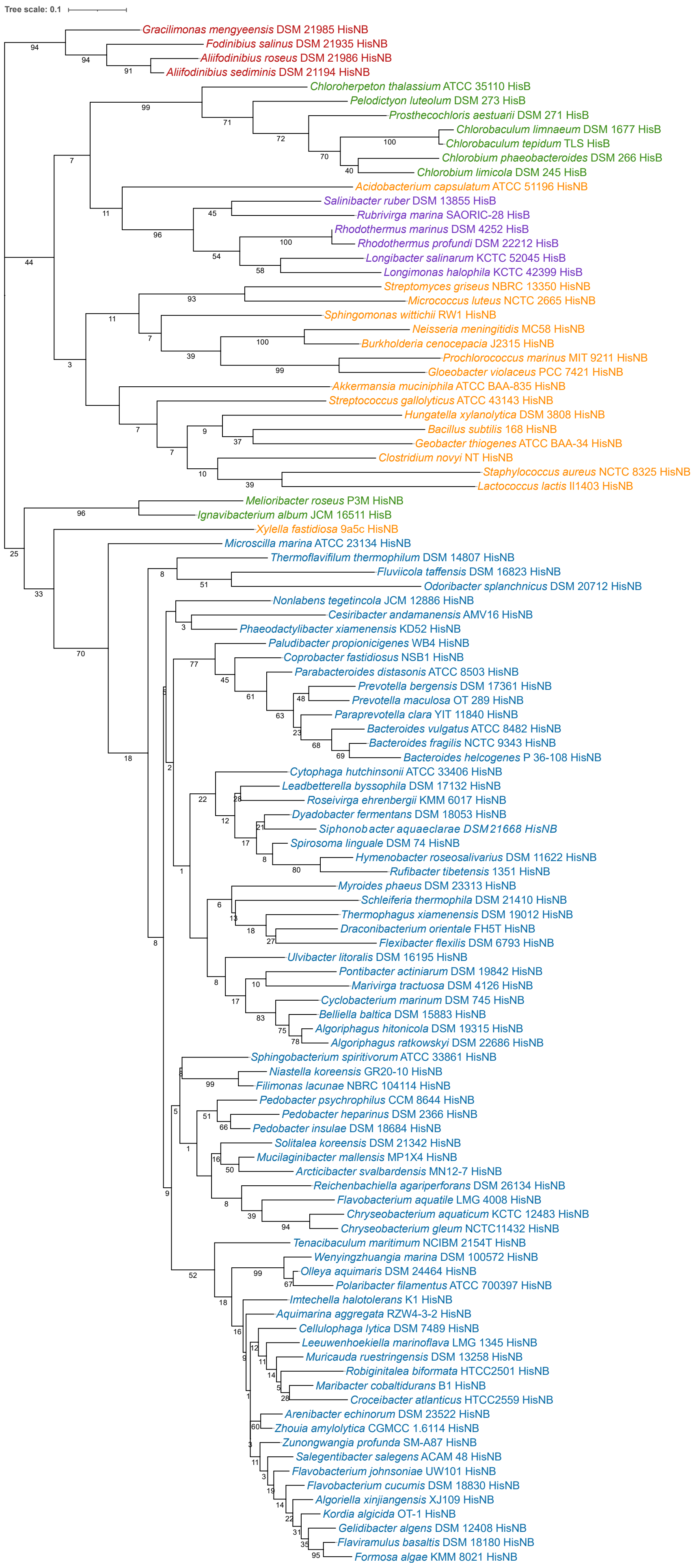

Figure S6. Phylogenetic tree based on the HisB amino acid sequences of the 91 superphylum organisms and 17 outgroups. After the multi-alignment trimming, 314 positions have been considered for the phylogenetic tree construction. Organisms reported in orange are the outgroups. Those in blue are Bacteroidetes, in green Chlorobi, in red Balneolota, and in violet Rhodothermota.

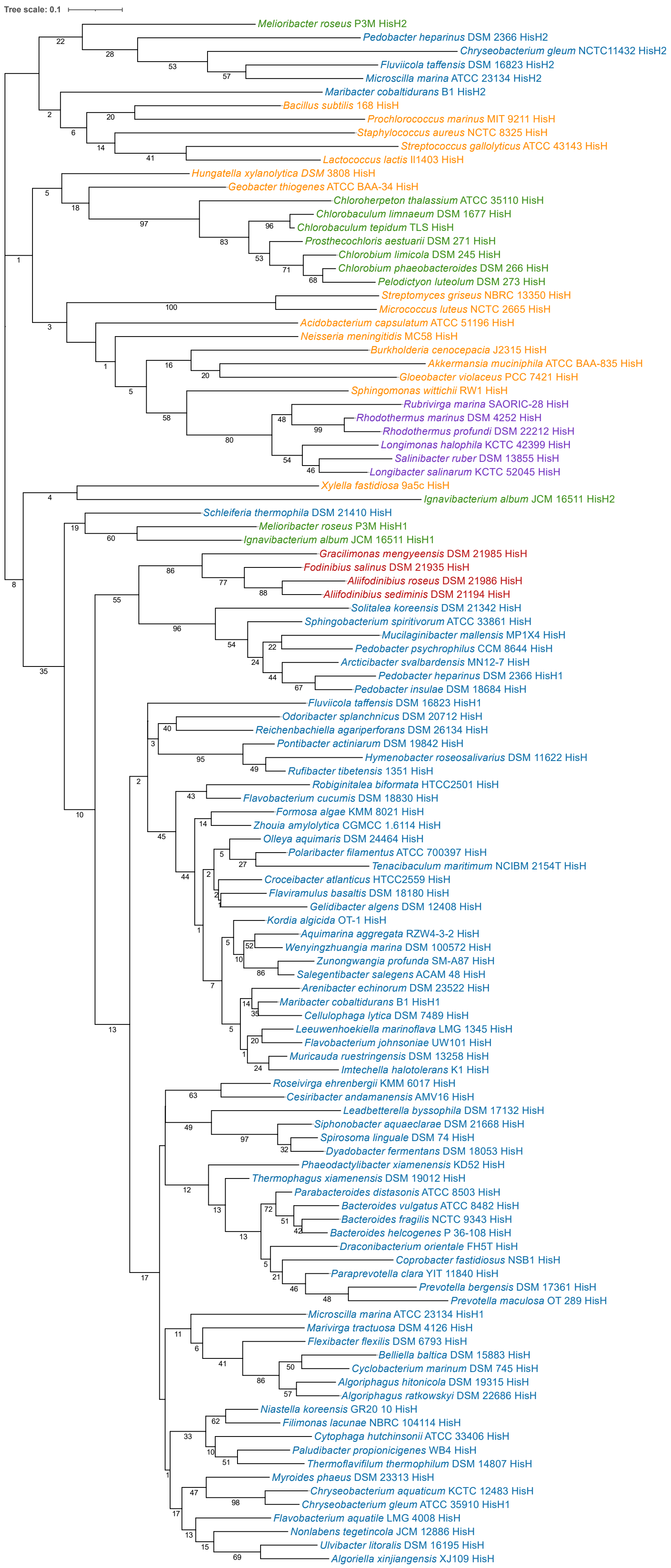

Figure S7. Phylogenetic tree based on the HisH amino acid sequences of the 91 superphylum organisms and 17 outgroups. After the multi-alignment trimming, 137 positions have been considered for the phylogenetic tree construction. Organisms reported in orange are the outgroups. Those in blue are Bacteroidetes, in green Chlorobi, in red Balneolota, and in violet Rhodothermota.

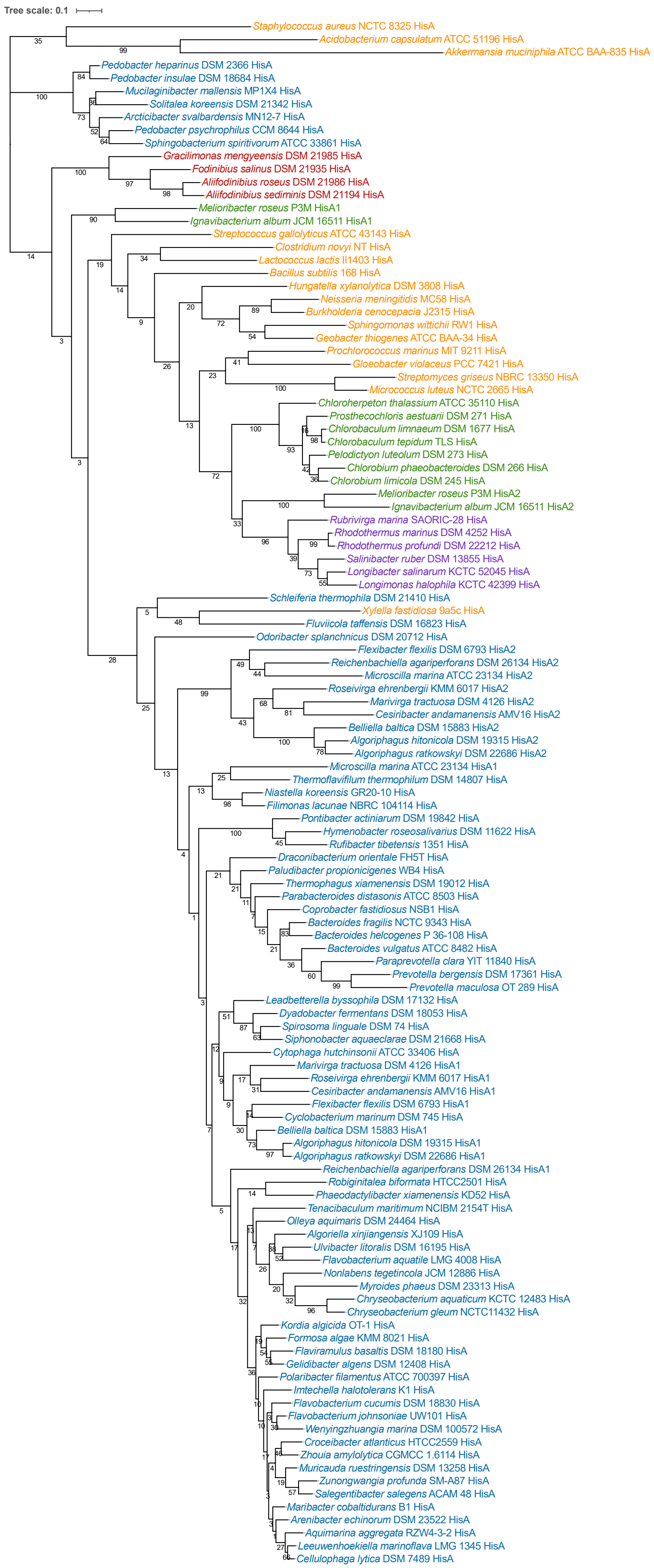

Figure S8. Phylogenetic tree based on the HisA amino acid sequences of the 91 superphylum organisms and 17 outgroups. After the multi-alignment trimming, 185 positions have been considered for the phylogenetic tree construction. Organisms reported in orange are the outgroups. Those in blue are Bacteroidetes, in green Chlorobi, in red Balneolota, and in violet Rhodothermota.

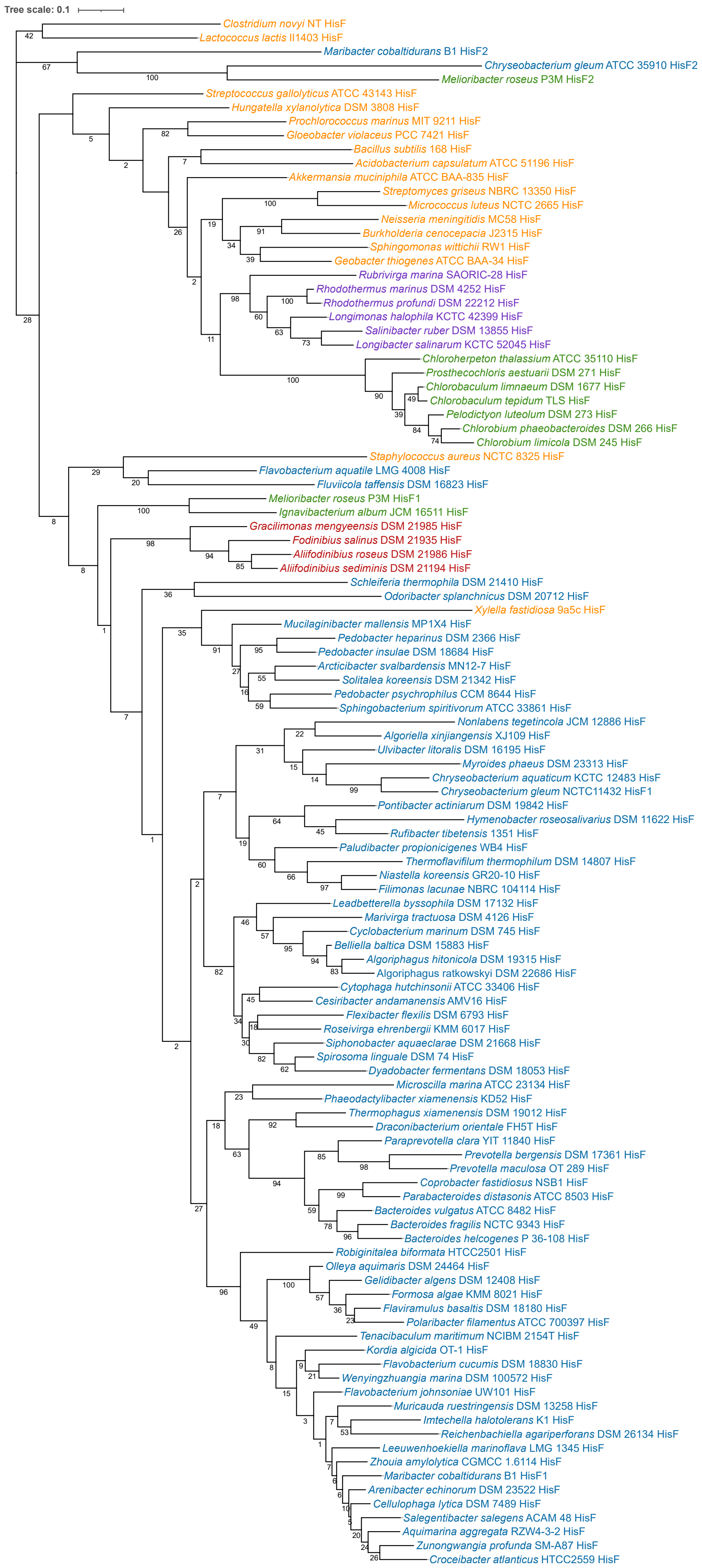

Figure S9. Phylogenetic tree based on the HisF amino acid sequences of the 91 superphylum organisms and 17 outgroups. After the multi-alignment trimming, 250 positions have been considered for the phylogenetic tree construction. Organisms reported in orange are the outgroups. Those in blue are Bacteroidetes, in green Chlorobi, in red Balneolota, and in violet Rhodothermota.

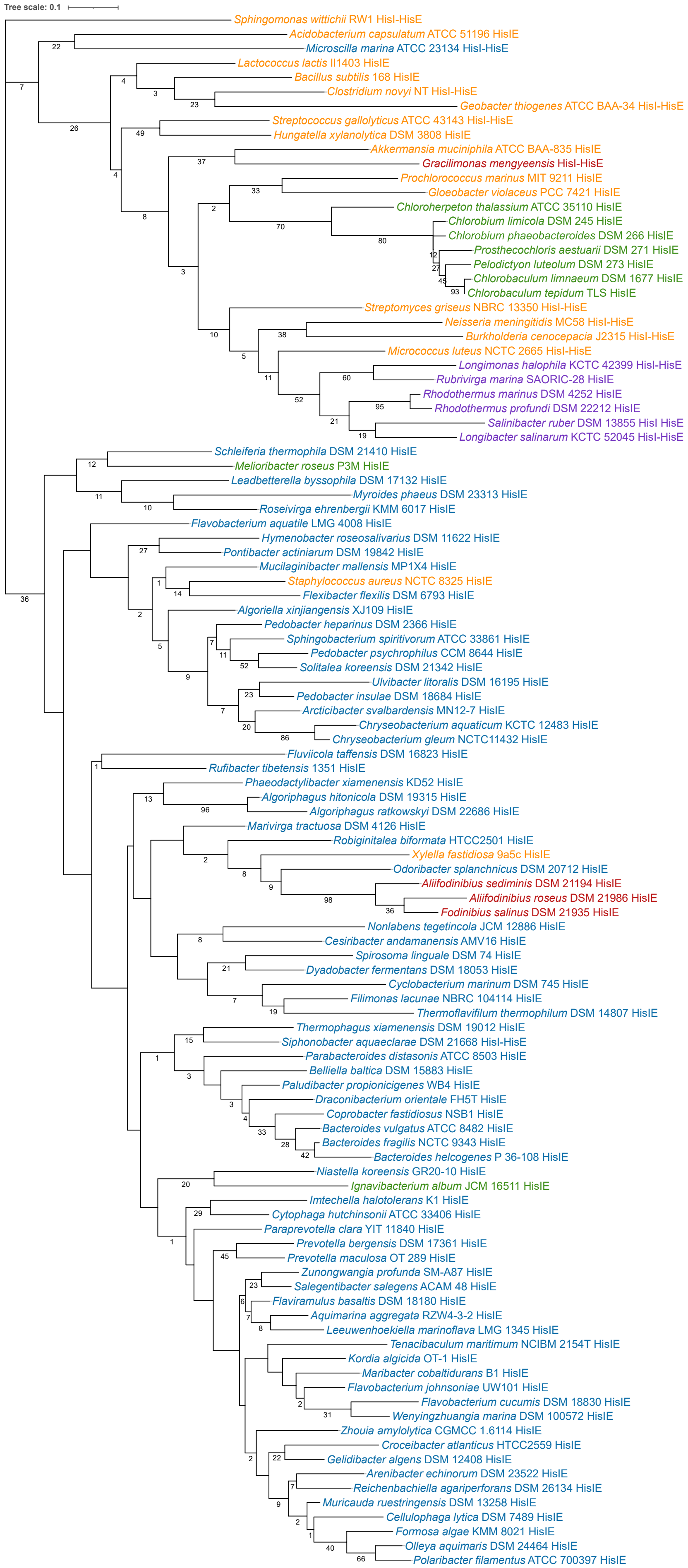

Figure S10. Phylogenetic tree based on the HisIE amino acid sequences of the 91 superphylum organisms and 17 outgroups (HisI and HisE sequences have been concatenated for those organisms with separated genes). After the multi-alignment trimming, 145 positions have been considered for the phylogenetic tree construction. Organisms reported in orange are the outgroups. Those in blue are Bacteroidetes, in green Chlorobi, in red Balneolota, and in violet Rhodothermota.

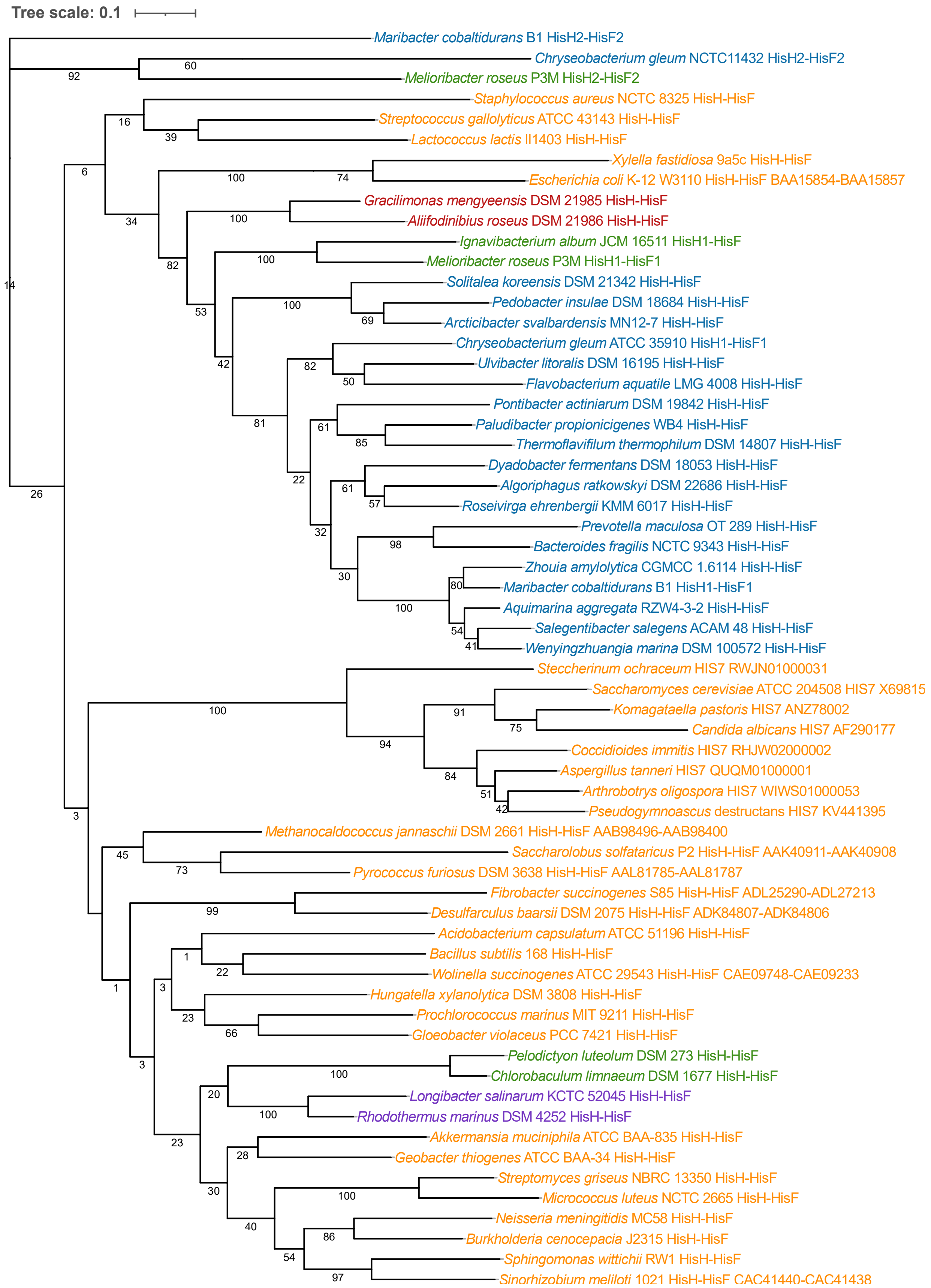

Figure S11. Phylogenetic tree constructed using the concatenated sequences of HisH and HisF or HIS7 of some members of the superphylum (19 Bacteroidetes, 4 Chlorobi, 2 Balneolota and 2 Rhodothermota) and outgroups representative of Bacteria (21), Archaea (3) and Eukarya (8). For those outgroups not reported in Table S2 (Additional file S1), the accession numbers of HisH and HisF or HIS7 proteins have been reported in the tree. After the multi-alignment trimming, 426 positions have been considered for the phylogenetic tree construction. Organisms reported in orange are the outgroups. Those in blue are Bacteroidetes, in green Chlorobi, in red Balneolota, and in violet Rhodothermota.
